# Developing a national patient safety plan in Guatemala

**DOI:** 10.26633/RPSP.2019.64

**Published:** 2019-07-31

**Authors:** Randall Lou-Meda, Sindy Méndez, Erwin Calgua, Mónica Orozco, Bria J. Hall, Natalie Fahsen, Brad M. Taicher, Joseph P. Doty, Julio García Colindres, Carlos Soto Menegazzo, Henry E. Rice

**Affiliations:** 1 Pediatric Nephrology Unit/Fundanier, Roosevelt Hospital Pediatric Nephrology Unit/Fundanier, Roosevelt Hospital Guatemala CityGuatemala Pediatric Nephrology Unit/Fundanier, Roosevelt Hospital, Guatemala City, Guatemala.; 2 University of San Carlos of Guatemala University of San Carlos of Guatemala Guatemala CityGuatemala University of San Carlos of Guatemala, Guatemala City, Guatemala.; 3 Duke Global Health Institute Duke Global Health Institute DurhamNorth Carolina United States of America Duke Global Health Institute, Durham, North Carolina, United States of America.; 4 FUNDEGUA FUNDEGUA Guatemala CityGuatemala FUNDEGUA, Guatemala City, Guatemala.; 5 Department of Orthopedics Department of Orthopedics Duke University Medical Center DurhamNorth Carolina United States of America Department of Orthopedics, Duke University Medical Center, Durham, North Carolina, United States of America.; 6 Guatemala Ministry of Public Health and Social Assistance Guatemala Ministry of Public Health and Social Assistance Guatemala CityGuatemala Guatemala Ministry of Public Health and Social Assistance, Guatemala City, Guatemala.

**Keywords:** Quality assurance, health care, patient safety, medical errors, health planning, Guatemala, Garantía de la calidad de atención de salud, seguridad del paciente, errores médicos, planificación en salud, Guatemala, Garantia da qualidade dos cuidados de saúde, segurança do paciente, erros médicos, planejamento em saúde, Guatemala

## Abstract

**Objective.:**

Patient safety is challenging for health systems around the world, particularly in low-and middleincome countries such as Guatemala. The goal of this report is to summarize a strategic planning process for a national patient safety plan in Guatemala.

**Methods.:**

This strategic planning process involved multiple stakeholders, including representatives of the Guatemala Ministry of Health and Social Assistance, medical leadership from across the public health system, and academic experts from Guatemala and the United States of America. We used mixed methods (quantitative and qualitative surveys) and a nominal group technique at a national symposium to prioritize patient safety challenges across Guatemala, and subsequent meetings to develop a national patient safety plan.

**Results.:**

This national patient safety plan outlines four domains to advance patient safety across the public hospital system over a five-year period in Guatemala: leadership and governance, training and awareness, safety culture, and outcome metrics. For each domain, we developed a set of goals, activities, outputs, and benchmarks to be overseen by the Ministry of Health.

**Conclusions.:**

With this national patient safety plan, Guatemala has made a long-term commitment to improving patient safety across the public hospital system of Guatemala. Future efforts will require its extension to all levels of the Guatemalan health system.

## RESUMEN

**Objetivo.:**

La seguridad del paciente constituye un reto para los sistemas de salud de todo el mundo, particularmente en los países de ingresos medianos y bajos como Guatemala. El objetivo del presente informe es resumir el proceso de planificación estratégica para elaborar un plan nacional sobre seguridad del paciente en Guatemala.

**Métodos.:**

En el proceso de planificación estratégica participaron varios interesados directos, entre los cuales se encontraron representantes del Ministerio de Salud Pública y Asistencia Social de Guatemala, autoridades médicas de todo el sistema de salud pública, así como expertos académicos de Guatemala y Estados Unidos de América. Para establecer las prioridades en cuanto a los retos que deben abordarse en Guatemala en materia de seguridad del paciente se utilizó un método mixto (encuestas cuantitativas y cualitativas) y la técnica de grupo nominal en un simposio nacional, y posteriormente se realizaron reuniones para elaborar un plan nacional sobre la seguridad del paciente.

**Resultados.:**

En el plan nacional sobre la seguridad del paciente se presentan cuatro ámbitos en los cuales se puede impulsar la seguridad del paciente en todos los hospitales públicos de Guatemala en un período de cinco años: liderazgo y gobernanza, capacitación y concientización, cultura de seguridad y métodos para medir los resultados. Se creó un conjunto de metas, actividades, resultados y puntos de referencia para cada ámbito, que estarán bajo la supervisión del Ministerio de Salud.

**Conclusiones.:**

Con este plan nacional sobre la seguridad del paciente, Guatemala ha adquirido el compromiso a largo plazo de mejorar la seguridad del paciente en los hospitales públicos del país. Las iniciativas que se emprendan en el futuro requerirán que se extienda a todos los niveles del sistema de salud guatemalteco.

## RESUMO

**Objetivo.:**

A segurança do paciente representa um desafio aos sistemas de saúde em todo o mundo, sobretudo nos países de baixa e média renda como a Guatemala. O presente tem o objetivo de descrever sucintamente o processo de planejamento estratégico para um plano nacional de segurança do paciente na Guatemala.

**Métodos.:**

Este processo de planejamento estratégico contou com a participação de diversos interessados diretos, como representantes do Ministério da Saúde e Assistência Social da Guatemala, autoridades médicas da área de saúde pública e especialistas acadêmicos da Guatemala e dos Estados Unidos. Métodos combinados (pesquisas quantitativas e qualitativas) e técnica de grupo nominal foram usados em um simpósio nacional para priorizar os desafios em segurança do paciente na Guatemala e reuniões subsequentes foram realizadas para elaborar o plano nacional de segurança do paciente.

**Resultados.:**

No plano nacional de segurança do paciente, são definidos quatro domínios para fazer avançar o processo de segurança do paciente no sistema hospitalar público em um período de cinco anos: liderança e governança; capacitação e conscientização; cultura de segurança e métricas para mensuração dos resultados. Um conjunto de metas, atividades, resultados e parâmetros de referência foi elaborado para cada domínio, a ser supervisado pelo Ministério da Saúde.

**Conclusões.:**

Com este plano nacional de segurança do paciente, a Guatemala assumiu um compromisso de longo prazo de melhorar a segurança do paciente no sistema hospitalar público. Esforços devem ser empreendidos para expandir o plano a todos os níveis do sistema de saúde da Guatemala.

Patient safety is increasingly recognized in the global health agenda as a central component of a high-quality health system (1, 2). The social, health, and economic consequences of poor health care highlight the need for systems that target the identification, prevention, and mitigation of medical errors. Quality improvement (QI) can strengthen health care delivery, improve health sector performance, and accelerate attainment of health-related Sustainable Development Goals (SDGs) through efforts to measure health care quality and to improve the delivery of high-quality health care (1, 3).

Medical errors are particularly hazardous in low- and middle-income countries (LMICs) such as Guatemala, where the risks of health care–related complications far exceed those in high-income countries (4, 5). Specific to Latin America, the Latin American Study of Adverse Events (IBEAS) (sometimes called the Iberoamerican Study of Adverse Events) found that, overall, 10.5% of the patients surveyed in 58 hospitals in Argentina, Colombia, Costa Rica, Mexico, and Peru had experienced a medical error, mostly related to health care–associated infections (HAIs), surgical procedures, patient management, and nursing care (6). Although concerns about patient safety in LMICs have driven the adoption of tools such as pre-procedural checklists, drug safety systems, and process standardization programs, the implementation of comprehensive safety programs across Latin America and the Caribbean remains limited (5, 7).

Health systems around the world have adopted common strategies to promote patient safety, including enhanced systems to measure medical errors, use of performance indicators, and implementation of evidence-based clinical guidelines (2). Several Latin American countries have led patient safety efforts, including Brazil, Guatemala, and Mexico (8-11). However, the successful implementation of comprehensive national patient safety programs in Latin America remains limited.

## GUATEMALA CONTEXT

Guatemala’s health system faces many internal and external challenges (12, 13). Similar to other Latin American countries, Guatemala has a fragmented health care system, with care provided within the public sector through the Ministry of Public Health and Social Assistance (MSPAS) and the Guatemalan Social Security Institute (IGSS), as well as through the private sector. Guatemala ranks near the lowest among world countries in terms of efficiency of health spending (5, 14, 15), and has some of the poorest health metrics across Latin America, such as with the under-5 mortality rate and immunization coverage (16). In 2014, 60% of the population (mainly indigenous people) still lived below the national poverty line of about US$ 3.50 daily (17). Social spending in Guatemala is among the lowest in Latin America, with just 2% of 2015 gross domestic product allocated to public health (17).

Despite several programs to improve health outreach in the public health system, particularly in rural areas and in primary care, wide disparities in health care and outcomes remain across Guatemala. For example, the North and Northwest regions have the country’s highest maternal mortality ratio (MMR), which is a common metric of health care quality, as well as the highest prevalence of stunting (12). Most of the regions with the highest MMR levels also have the lowest health spending per capita (12). Both patients and health care personnel have expressed concerns about health care quality in Guatemala, which has led to new programs to improve patient safety (11, 18).

Within this context, a working group composed of MSPAS representatives, medical and hospital leaders, academic experts from the University of San Carlos of Guatemala (USAC) and Duke University (Durham, North Carolina, United States of America), and local nongovernmental organization personnel initiated a strategic planning process to improve patient safety. Following a kickoff symposium in October 2018, this interdisciplinary group of stakeholders developed a national patient safety plan, which was endorsed by the Ministry of Health in April 2019.

The goals of this commentary are to summarize this strategic planning process, identify priorities for patient safety, and develop a framework to improve patient safety across the public health system in Guatemala.

## PATIENT SAFETY SYMPOSIUM

To initiate this strategic planning process, we first performed a stakeholder analysis at a symposium held in Guatemala City in October 2018. Invitees included hospital directors from all 38 public hospitals across the country, representatives from the MSPAS and from private hospitals, and academic leaders from Duke University and USAC. The final slate of attendees included 36 public hospital directors representing all 22 departments of Guatemala; 13 representatives from the MSPAS, including the vice minister of hospitals; 1 private hospital director; and 10 academic leaders from Duke University and USAC. The goals of this symposium were to: 1) identify challenges to patient safety within the Guatemalan public health system and 2) prioritize strategies to improve patient safety.

We collected data from public hospital directors (31 hospital directors completed full surveys, for a 86% response rate) using several methods. To identify challenges to patient safety, we used quantitative surveys and guided group discussions. Participants were first asked to identify, discuss, and rank problems in patient safety at their home institution using a written survey. To prioritize interventions, we led focus group discussions using a nominal group technique to prioritize health care safety concerns (19). Through this technique, participants first selected which patient safety challenges they felt were most important to their institution. Participants presented their ideas using a group facilitator, and then all suggestions were discussed and prioritized by the entire group. The nominal group findings were triangulated with the written survey data to develop a final list of priority areas in patient safety and to propose intervention strategies. The Duke University Medical Center Institutional Review Board exempted this study from oversight.

## STAKEHOLDER ANALYSIS

Almost all (98%) of the symposium participants consider the Guatemala health system to have significant problems in patient safety and health care quality. There was wide variation among hospital leadership in regard to awareness and use of patient safety policies, although most directors cited a need to expand local patient safety systems. Most participants cited a lack of awareness of patient safety among health care staff, with only 27% of directors thinking that their staff had adequate knowledge about patient safety. Critical barriers included a lack of national health policy on patient safety, insufficient training in patient safety for health care personnel, and lack of staff awareness of the importance of patient safety. We triangulated data from focus group discussions and written surveys to develop a final ranking of national patient safety priorities (Table 1).

## STRATEGIES TO IMPROVE PATIENT SAFETY

Following data analysis, the working group collaborated over the next three months in developing short-term and longterm strategies to improve patient safety and health care quality as well as providing a framework for a national patient safety plan. This strategic planning process involved triangulation of quantitative survey data, evaluation of discussions from the symposium, and a series of meetings to drive consensus to prioritize interventions and strategies.

### Short-term interventions

There were four key high-ranking short-term interventions (< 12 months) to improve patient safety. The first concerned leadership and organization. A national Patient Safety Leadership Committee composed of multiple stakeholders should be developed to establish priorities for patient safety efforts across the public health system. Stakeholders should include public and private medical sector leaders, government officials, patients, and national and international experts in patient safety. The second intervention dealt with training, with training core staff across the public health system at the local level to lead interdisciplinary patient safety teams. The third short-term invention focused on safety culture and awareness, with the use of data-driven tools to promote safety culture and awareness among health care staff and the general population. The fourth concerned research, with the expansion of research capacity to assess patient safety, evaluation of the economic impact of patient safety, and tackling of implementation challenges

**TABLE 1.** Barriers to patient safety in Guatemala, as ranked by participants in an October 2018 symposium in Guatemala City, in order of importance (with 1 representing the most critical and 9 the least critical problem)

Ranked topic	Mean
1. National policy: lack of national policies defining patient safety standards	2.95
2. Knowledge and learning: insufficient training on patient safety for health care personnel	3.98
3. Awareness: lack of awareness of health professionals and general population of patient safety	4.61
4. Safety culture: biases in regards to patient safety (e.g., “good physicians never make mistakes,” tendency to place personal blame for mistakes, errors can be eliminated through punitive measures)	4.74
5. Health systems: lack of patient safety infrastructure (e.g., trained personnel, committees, etc. to address health care–associated infections, medication safety)	5.13
6. Resources: lack of incentives for adoption of patient safety systems	5.18
7. Research: lack of research on patient safety problems in Guatemala	5.66
8. Data collection systems: limited technologies to collect information on medical errors	6.32
9. Key performance indicators: lack of appropriate key performance indicators to assess health services	6.67

**TABLE 2.** Domains of national patient safety plan for Guatemala developed using a strategic planning process in 2018-2019, with goals, activities, outputs, and benchmarks

Domain	Goal(s)	Activity	Output(s)	Benchmark
Leadership and governance	A. Develop national patient safety committee	Set national strategy to improve patient safety across Guatemala	i. Engage stakeholders from across health system	Dissemination of plan across health system
	B. Identify local patient safety champions	Identify patient safety officers in their institution	i. Local patient safety teams and operations	Champions appointed in 75% of hospitals
Training and awareness	A. Train local leaders to lead safety teams	Training conducted for patient safety leaders	i. Enrollment in patient safety training programs	Enroll trainees from 75% of sites
	B. Promote staff education and awareness	Educational initiatives for health care staff	i. Education and sensitization campaigns	Workshops in 75% of sites
	C. Integrate patient safety and leadership into curricula	Standardize patient safety curriculum for health-related training	i. Professional schools will include training in patient safety	Integrate patient safety concepts in curriculum
Safety culture	A. Improve patient safety through safety culture assessments	Institutional safety teams to deploy safety culture assessments	i. Safety culture metrics ii. Local safety and quality interventions	Improve safety culture by 10% in 75% of hospitals
Outcome metrics and reporting	A. Define standard outcome metrics and safety indicators	Set national standards for indicators to measure health care quality	i. Adoption of patient safety indicators	Publication of safety standards and metrics
	B. Develop medical error reporting systems	Implement safety reporting systems to track medical errors	i. Integrate medical errors into national health information systems	Error reporting systems in 75% of sites

### Medium-term and long-term interventions

The proposed intervention strategies for the medium to long term (one to five years) covered four areas. The first was national policy development, including developing a national policy on patient safety and health care quality, as well as defining standards, priorities, and framework for patient safety. The second area was informatics, in terms of developing and supporting infrastructure for adverse event reporting. The third was training, with the expansion of patient safety education programs for health care staff, and the integration of patient safety and leadership content into medical education. The fourth area was harmonization, through the unification of regional and national quality improvement and patient safety initiatives.

## NATIONAL PATIENT SAFETY PLAN

The strategic working group used this information to develop a national patient safety plan. We first developed a framework to support patient safety across the public hospital system based on principles of several international patient safety organizations and of the World Health Organization (WHO) (2, 20). Use of an established framework allowed us to ensure that patient safety indicators were measured along the continuum of care from service readiness to service delivery. Given the heterogeneity in Guatemala culture and health care settings, our intent was not to create a rigid framework, but to prioritize indicators that are relevant to the implementation of patient safety programs across a wide range of health care settings.

The national patient safety plan outlines four domains to guide patient safety initiatives: leadership and governance, training and awareness, safety culture, and outcome metrics (Table 2). For each of these domains, we developed a set of goals, activities, outputs, and benchmarks to guide implementation over a five-year period. These elements were developed through extraction from existing research, guidelines, and WHO policy recommendations (21). This plan was approved by the Ministry of Health in April 2019. The plan is intended to begin over the following 12-24 months, with interval assessment of program success. Depending on program success, the plan may be used to guide national patient safety efforts for other elements of the health system, budget development, and alignment with other health priorities.

We recognize several limitations to this national patient safety plan. First, as this program is focused towards patient safety in public hospitals, not all stakeholders in the Guatemalan health system were included in the planning process, particularly from primary care and rural settings. To equitably support patient safety across the Guatemalan health system, other groups should be included as this program is further developed, including with representation from the IGSS, the Health Care Unit of Indigenous Peoples and Interculturality (UASPII), National Reproductive Health Program (PNSR), General Directorate of the Comprehensive Health Care System (DGSIAS), Health Areas Directorates (DAS), Ombudsman’s Office for Indigenous Women (DEMI), and the Presidential Secretariat for Women (SEPREM). Second, we recognize that Guatemala has some of the lowest levels of public health investment in Latin America, suggesting that increased government investment may be required to implement this patient safety agenda (17, 22). Third, we did not include any cost-benefit or economic analysis of this program, which is essential to support ongoing investment in this program. Finally, we recognize that implementation of patient safety programs remains challenging around the world. This program will require formal implementation analysis to identify barriers and facilitators to sustained adoption and integration of patient safety programs into existing health processes.

## DISCUSSION

This national safety plan represents Guatemala’s long-term commitment to improving health care quality and patient safety, and will be a core element of the MSPAS’s operations. This national patient safety plan is a necessary key—but only first—step towards driving improvements in patient safety. Although the national patient safety plan is one indicator that the building blocks are in place to provide high-quality care, a national strategy alone has limited capacity to promote action. To augment these policy efforts, we advocate efforts to promote a “just culture,” or support a safety culture that values transparency, communication, and investment in trained personnel and systems to promote continuous quality improvement (11).

Standardized, locally applicable quality indicators are required to monitor progress in health care quality (1). Quality indicators are used around the world for performance benchmarking, including in Guatemala (16, 23, 24). We recognize that in Guatemala, incentive systems that link health system regulations and/or accreditation to performance indicators may be required. In line with other Government health priorities, we encourage health reforms to address rising health care costs. In terms of patient safety, these reforms may help alleviate the problem of inequitable focus on patient safety across multiple levels of health care between the public and private sectors (12).

Our experience may be helpful for other countries that are developing national safety plans, as all countries face complex challenges in patient safety. A national patient safety plan should be contextualized to a country’s resources and health delivery challenges. These kinds of plans provide a framework to define the role of government and other stakeholders in assuring patient safety across the health system; clarify responsibilities and relationships; and identify incentives to support optimal performance in the provision of high-quality care. We encourage an implementation framework to embed patient safety programs in existing health delivery systems. Finally, we emphasize the need for education of health care providers in quality improvement and patient safety, as well as support for formal training of local experts to lead patient safety efforts.

### Author contributions.

Randall Lou-Meda: study design and manuscript preparation; Sindy Méndez: data collection; Erwin Calgua: study design; Mónica Orozco: data analysis; Bria J. Hall: study design, data collection, and manuscript preparation; Natalie Fahsen: data collection; Brad M. Taicher: study design; Joseph P. Doty: study design; Julio García Colindres: study design; Carlos Soto Menegazzo: study design; and Henry E. Rice: study design, data collection, and manuscript preparation. All the authors reviewed and approved the final version.

### Acknowledgments.

We thank the FUNDEGUA foundation, Fundanier foundation, University of San Carlos of Guatemala, Duke Global Health Institute, and Guatemala Ministry of Public Health and Social Assistance (Ministerio de Salud Pública y Asistencia Social) for their support. No funding agency influenced, in any way, the design, data collection, analysis, writing, or decision to publish these results.

## Disclaimer.

The authors hold sole responsibility for the views expressed in the manuscript, which may not necessarily reflect the opinion or policy of the *RPSP/PAJPH* and/or PAHO.
